# Biochemical studies of the multicopper oxidase (small laccase) from *Streptomyces coelicolor* using bioactive phytochemicals and site-directed mutagenesis

**DOI:** 10.1111/1751-7915.12068

**Published:** 2013-07-01

**Authors:** Mohammed Sherif, Debbie Waung, Bihter Korbeci, Valentina Mavisakalyan, Robert Flick, Greg Brown, Mamdouh Abou-Zaid, Alexander F Yakunin, Emma R Master

**Affiliations:** 1 Department of Chemical Engineering and Applied Chemistry, University of Toronto200 College Street, Toronto, ON, M5S 3E5, Canada; 2 Banting and Best Department of Medical Research, University of TorontoToronto, ON, M5G 1L6, Canada; 3 Great Lakes Forestry Centre1219 Queen Street East, Sault Ste. Marie, ON, P6A 2E5, Canada

## Abstract

Multicopper oxidases can act on a broad spectrum of phenolic and non-phenolic compounds. These enzymes include laccases, which are widely distributed in plants and fungi, and were more recently identified in bacteria. Here, we present the results of biochemical and mutational studies of small laccase (SLAC), a multicopper oxidase from *Streptomyces coelicolor* (SCO6712). In addition to typical laccase substrates, SLAC was tested using phenolic compounds that exhibit antioxidant activity. SLAC showed oxidase activity against 12 of 23 substrates tested, including caffeic acid, ferulic acid, resveratrol, quercetin, morin, kaempferol and myricetin. The kinetic parameters of SLAC were determined for 2,2′-azino-bis(3-ethylbenzthiazoline-6-sulphonic acid), 2,6-dimethoxyphenol, quercetin, morin and myricetin, and maximum reaction rates were observed with myricetin, where *k_cat_* and *K_m_* values at 60°C were 8.1 (± 0.8) s^−1^ and 0.9 (± 0.3) mM respectively. SLAC had a broad pH optimum for activity (between pH 4 and 8) and temperature optimum at 60–70°C. It demonstrated remarkable thermostability with a half-life of over 10 h at 80°C and over 7 h at 90°C. Site-directed mutagenesis revealed 17 amino acid residues important for SLAC activity including the 10 His residues involved in copper coordination. Most notably, the Y229A and Y230A mutant proteins showed over 10-fold increase in activity compared with the wild-type SLAC, which was correlated to higher copper incorporation, while kinetic analyses with S929A predicts localization of this residue near the *meta*-position of aromatic substrates.

**Funding Information** Funding for this research was provided by the Government of Ontario for the project ‘FFABnet: Functionalized Fibre and Biochemicals’ (ORF-RE-05-005), and the Natural Sciences and Engineering Research Council of Canada.

## Introduction

Laccases (EC 1.10.3.2; benzenediol : oxygen oxidoreductase) belong to the diverse family of multicopper oxidases (MCOs), which are characterized by having four copper (II) ions per domain that mediate one-electron oxidation of four substrate molecules with concomitant reduction of molecular oxygen to water. These enzymes are widely distributed in nature, and to date, more than 100 laccases have been isolated and characterized (Hildén *et al*., 2009; Giardina *et al*., 2010). Most of these laccases were isolated from fungi and shown to participate in the depolymerization of lignin (Baldrian, 2006). However, genomic and metagenomic analyses increasingly show that MCOs with laccase activity are also widespread among bacteria (Beloqui *et al*., 2006; Kellner *et al*., 2008; Ferrer *et al*., 2010; Ausec *et al*., 2011). While bacterial MCOs are thought to play a role in sporulation, pigmentation and metal homeostasis, these enzymes have also been implicated in bacteria-mediated lignin degradation (Claus, 2003; Bugg *et al*., 2011).

The oxidative versatility of MCOs including laccases has been exploited in a wide variety of applications including the delignification of wood, increasing the strength of mechanical pulp and compatibilization of plant fibre used in biocomposite materials (Widsten and Kandelbauer, 2008; Mikolasch and Schauer, 2009). MCOs have also been applied in fuel cells and to remediate industrial effluents (Mayer and Staples, 2002; Rodriguez Couto and Toca Herrera, 2006; Smolander *et al*., 2007). Moreover, recent reviews discuss the value of MCOs in organic syntheses (Riva, 2006; Kunamneni *et al*., 2001; Jeon *et al*., 2012). For example, the ability of MCOs to polymerize quercetin and other plant flavonoids has been proposed as a route to synthesizing bioactive compounds with nutraceutical benefits (Desentis-Mendoza *et al*., 2006; Ponzoni *et al*., 2007; Uzan *et al*., 2011).

Despite the range of processes that can harness laccase and other MCO activity, industrial-scale application of these enzymes for the production of commodity products like pulp and biofuels are limited in part by the availability of low cost, biodegradable mediators of the enzyme activity (Camarero *et al*., 2007; Cañas and Camarero, 2010). Currently, 2,2′-azino-bis(3-ethylbenzthiazoline-6-sulphonic acid) (ABTS) is commonly used to mediate laccase activity. Alternative plant-derived phenolic compounds, including syringaldehyde, ferulic acid and *p*-coumaric acid, can also mediate laccase activity in some cases (Camarero *et al*., 2008), although cost-effective isolation of sufficient quantities can be challenging.

Large-scale application of laccases and other MCOs is also challenged by the cost of enzyme production, as well as enzyme inhibition at typical industrial process conditions (Kunamneni *et al*., 2001). While most laccases now in use were isolated from mesophilic fungi, bacterial MCOs with laccase activity could present a source of low-cost enzymes (Santhanam *et al*., 2011). In addition to being readily expressed in recombinant hosts and purified, thermostable MCOs from bacteria may represent particularly robust industrial catalysts.

Bacterial MCOs with laccase activity that have been biochemically characterized to date include: CotA from *Bacillus subtilis* (Hullo *et al*., 2001; Martins *et al*., 2002), CueO and PcoA from *Escherichia coli* (Grass and Rensing, 2001; Huffman *et al*., 2002), EpoA from *Streptomyces griseus* (Endo *et al*., 2003), CumA and CopA from *Pseudomonas* species (Brouwers *et al*., 1999; Solano *et al*., 2001), BCO from *Nitrosomonas europaea* (Lawton *et al*., 2009) and the small laccase (SLAC) from *Streptomyces coelicolor* (Machczynski *et al*., 2004). Protein structures are also available for CotA (Enguita *et al*., 2003), CueO (Li *et al*., 2007), BCO (Lawton *et al*., 2009) and SLAC (Skálová *et al*., 2009), and reveal that CotA and CueO are three domain proteins, whereas BCO is a trimer of type-C two-domain proteins and SLAC is a trimer of type-B two-domain proteins (Nakamura and Go, 2005).

As was previously reported, the ease of production in *E. coli*, thermostability and reported activity on 2,6-dimethoxyphenol (DMP) at alkaline pH, mean that SLAC could be an ideal candidate for numerous biotechnology applications, including organic syntheses (Machczynski *et al*., 2004). Accordingly, in the present study, SLAC was selected for detailed biochemical characterization using a broad range of compounds, including alternative mediators and bioactive phytochemicals. The availability of the SLAC structure also facilitated site-directed mutagenesis, which was performed to confirm key residues involved in copper binding, and to predict those that could be modified to alter the reactivity or substrate selectivity of this enzyme. Finally, since microaerobic cultivation conditions were previously shown to promote the incorporation of copper ions by CotA when recombinantly expressed in *E. coli* (Durão *et al*., 2008a), the effect of cultivation condition on the yield and specific activity of SLAC was evaluated.

## Results and discussion

### Effect of cultivation conditions on SLAC activity

SLAC was overexpressed in *E. coli* and affinity purified to over 95% homogeneity. Concentrated preparations of SLAC (∼30 mg ml^−1^) from both standard and microaerobic cultivation conditions had the characteristic dark blue colour indicative of Cu^2+^ incorporation (Fig. S1).

Importantly, the copper content of purified MCOs is often incomplete and depends on the cultivation medium, temperature and oxygen concentration (Galli *et al*., 2004; Durão *et al*., [Bibr b13],[Bibr b14]). Therefore, as previously done for CotA (Durão *et al*., 2008a), SLAC was prepared from microaerobic cultivations [growing the *E. coli* overnight at 16°C without shaking after induction with isopropyl-beta-D-1-thiogalactopyranoside (IPTG)] in an effort to increase the content of copper ions in the purified enzyme. Indeed, it was found that microaerobic growth conditions increased the copper content in SLAC from 0.5 to 1.2 moles of Cu^2+^ per mole of enzyme, when compared with enzyme from standard cultivation conditions (with continuous shaking of cells). Increased copper content correlated with an increase in specific activity on 1 mM ABTS, from 110 ± 10 nmol min^−1^ mg^−1^ to 980 ± 30 nmol min^−1^ mg^−1^. These results confirm that similar to previous studies with CotA (Durão *et al*., 2008a; Mohammadian *et al*., 2010), microaerobic cultivation conditions promote the incorporation of Cu^2+^ in SLAC.

### SLAC stability and optimal reaction conditions

The optimal temperature of most fungal laccases is between 30 and 55°C, although some are as high as 80°C (Baldrian, 2006; Hildén *et al*., 2009). Similar to the recently characterized CotA from *B. pumilus*, SLAC exhibited maximum activity on ABTS at 70°C (Fig. S2). Stability in a broad range of pH conditions enhances the applied significance of MCOs, and SLAC retained between 50% and 60% activity after preincubation for 5 h at pH 7.0–10.0, and between 60% and 80% activity after preincubation for 5 h at pH 4.0–6.0 (Fig. S3).

Fungal laccases from *Pycnoporus* species are among the few known laccases that are reported to have a half-life (T_1/2_) of more than 1 h at temperatures above 60°C (Hildén *et al*., 2009). By comparison, bacterial MCOs with laccase activity generally show higher thermal stability. For example, the T_1/2_ of CotA at 80°C is over 100 min (Martins *et al*., 2002), and the T_1/2_ of an MCO from *Streptomyces lavendulae* is 100 min at 70°C (Suzuki *et al*., 2003). SLAC isolated from cultivations grown using standard conditions revealed remarkable thermal stability, having a T_1/2_ at 90°C of over 7 h, and T_1/2_ at 100°C of approximately 1 h (Table 1). Since the *S. coelicolor* cells have moderate optimal growth temperature [around 28°C (Bursy *et al*., 2008)], the thermostability of SLAC might be associated with the overall protein fold and oligomerization like in the *E. coli* and yeast pyrophosphatases (Ichiba *et al*., 1988). Notably, the thermostability of SLAC prepared from microaerobically grown cell was somewhat lower than that prepared from standard growth conditions (Table 1).

**Table 1:** Thermal stability of small laccase (SLAC) prepared using standard or microaerobic growth conditions

	Half-life (h)^a^	
Temperature (°C)	Standard growth	Microaerobic growth
80.1	>15 ± 0.7	11.4 ± 0.4
90.8	7.2 ± 1.3	1.6 ± 0.1
95.4	1.1 ± 0.4	0.22 ± 0.04
100.0	0.7 ± 0.2	0.13 ± 0.01

a Half-life (T_1/2_) values were measured using ABTS (pH 4.0). *n* = 3; errors indicate standard deviation.

### SLAC activity with low molecular weight mediators

**Figure 1:**
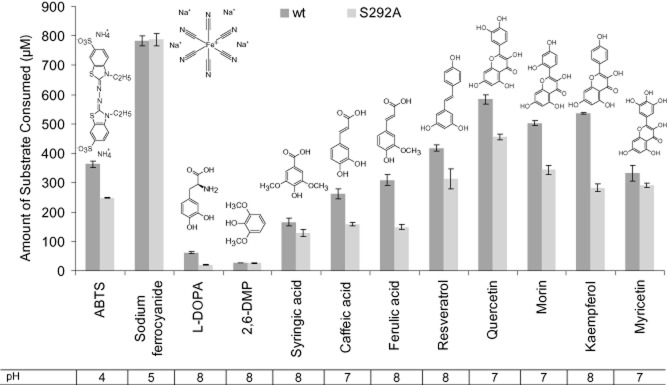
Substrate selectivity of small laccase (SLAC). Substrate oxidation by purified wild-type SLAC (dark grey bars) and variant S292A (light grey bars) on various substrates. Wild-type SLAC and the S292A variant were prepared using microaerobic growth conditions. Substrate depletion (μM) was measured after 20 min at 60°C and the optimal pH for the reaction. Initial substrate concentrations were 1000 μM. *n* = 3; errors indicate standard deviation.

To date, activity measurements of SLAC have been reported for only a handful of compounds, including 2,6-DMP (Machczynski *et al*., 2004). In the current study, SLAC was tested for oxidase activity against 23 different substrates, including natural, bioactive phenolic compounds. Consistent with results reported in Machczynski and colleagues (2004), SLAC prepared herein oxidized 2,6-DMP, Na_4_Fe(CN)_6_ and L-DOPA (Fig. 1). The current analysis also confirmed comparable substrate consumption by SLAC of resveratrol, quercetin, morin, myricetin and kaempferol relative to ABTS after 20 min of reaction (Fig 1). Overall, SLAC showed approximately 30 times higher substrate consumption towards the best substrate Na_4_Fe(CN)_6_ (783 ± 17 μM) as compared with the poorest substrate 2,6-DMP (27.0 ± 0.2 μM). SLAC activity on different substrates was pH-dependent, and optimum activities were detected between pH 4 and 8 (Fig. 1). The activity of SLAC on phenolic substrates was highest at alkaline pH, which is consistent with previous analyses of SLAC on 2,6-DMP (Machczynski *et al*., 2004), and the predicted influence of pH on the redox potential of phenol substrates (Xu, 1997).

New insight gained from this work as compared with the previous analysis of SLAC by Machczynski and colleagues (2004) includes the ability of SLAC to oxidize comparatively large phenolic substrates with multiple hydroxyl groups (like quercetin) in addition to smaller phenolics (like syringic acid). SLAC activity on polyaromatic compounds may be explained by the potential of unpaired electrons to delocalize after oxidation of the phenolic compound, leading to stabilized radical products. The electronic contribution of hydroxyl and/or methoxy substituents also generates phenoxy moieties that are more easily oxidized (Xu, 1996). This phenomenon could explain why SLAC oxidized caffeic acid and ferulic acid but not *p*-coumaric acid, which lacks a hydroxyl or methoxy substituent *ortho* to the single hydroxyl. Consistent with this hypothesis, reported redox potential values at pH 7.4 for *p*-coumaric acid, ferulic acid and caffeic acid are 0.66 V, 0.50 V and 0.36 V respectively (Jørgensen and Skibsted, 1998). Similarly, SLAC activity was observed on kaempferol but not apigenin. Apigenin differs from kaempferol by lacking a hydroxyl group adjacent to the carbonyl carbon (Fig. S4), and the reported redox potentials of kaempferol and apigenin at pH 7.4 is 0.39 V and 0.71 V respectively (Jørgensen and Skibsted, 1998).

### Kinetic analysis of SLAC

**Figure 2:**
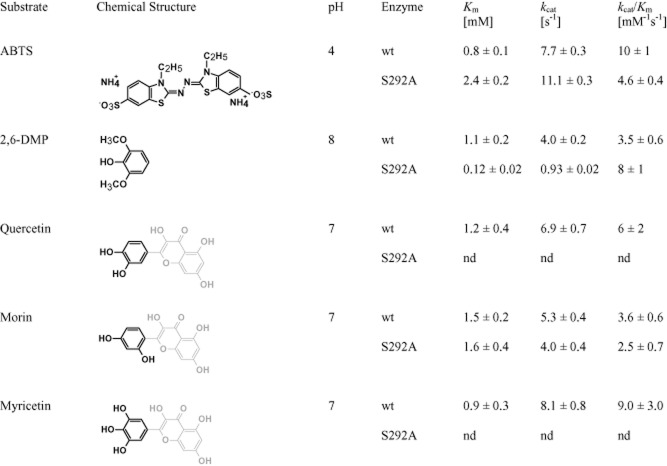
Kinetic parameters of wild-type small laccase (SLAC) and the S292A variant enzyme. Kinetic parameters were determined for wild-type (wt) SLAC on ABTS, 2,6-DMP, quercetin, morin and myricetin. Kinetic parameters were also determined for S292A on ABTS, 2,6-DMP, and morin, but could not be obtained for quercetin and myricetin since the apparent *K*_m_ of the enzyme for these substrates exceeded substrate solubility. Lighter shaded regions in quercetin, morin and myricetin molecules indicate identical chemical structures. *n* = 3; errors indicate standard deviation.

The kinetic parameters for SLAC were obtained at 60°C for ABTS, 2,6-DMP, quercetin, morin and myricetin (Fig. 2); it was not possible to determine kinetic parameters for Na_4_Fe(CN)_6_, L-DOPA, ferulic acid, resveratrol and kaempferol due to low solubility or high background absorbance of the compound at required substrate concentrations. The *K*_m_ values for SLAC were comparable to those reported for the CueO MCO from *E. coli* (YacK) (Kim *et al*., 2001); *K*_m_ and *k*_cat_ values for 2,6-DMP were also consistent with those reported by Machczynski and colleagues (2004). However, in this study, SLAC showed approximately two times lower *K*_m_ and three times lower *k*_cat_ on 2,6-DMP compared with the previous work (Machczynski *et al*., (2004) suggesting that the SLAC in this study has a stronger binding affinity but lower maximum turnover of this compound. This could be due to differences in copper content of SLAC produced in this work compared with earlier reports. For instance, since type-2 and type-3 copper ions are coordinated by neighbouring SLAC monomers, and that the coordination of type-2 and type-3 copper ions is predicted to promote the formation of the substrate-binding site (Skálová *et al*., 2009), copper content is likely to influence the folding and flexibility of the substrate cleft. The *K_m_* of SLAC for quercetin, morin and myricetin (1.2 ± 0.4 mM, 1.5 ± 0.2 mM and 0.9 ± 0.3 mM respectively) were comparable to those for ABTS and 2,6-DMP (0.8 ± 0.1 mM and 1.1 ± 0.2 mM respectively) (Fig. 2), indicating that SLAC effectively binds and transforms higher molecular weight phenolics and that activity is retained in 5% (v/v) of dimethyl sulfoxide required to solubilize the substrate. Notably, SLAC displayed highest *k*_cat_ values with myricetin (8.1 ± 0.8 s^−1^), which is consistent with the contribution of small, electron donating substitutions on electron transfer kinetics (Xu, 1996). Similar *k*_cat_ values with ABTS, quercetin and morin, but lower ABTS transformation after 20 min (Fig. 1), suggests products of ABTS oxidation might be inhibitory.

### Site-directed mutagenesis

The catalytic efficiency of MCOs and the range of substrates oxidized are determined partly by the redox potential of the type-1 copper that shuttles electrons from substrates to the trinuclear copper centre. Previous studies with CotA demonstrate the ability to manipulate the redox potential and catalytic efficiency of this enzyme through substituting amino acids involved in coordinating or stabilizing the type-I copper site (Durão *et al*., [Bibr b13],[Bibr b15]). In particular, replacing Met502 by leucine or phenylalanine increased the redox potential of CotA, while replacing L386 and I494 with alanine decreased the redox potential and catalytic efficiency of the enzyme.

The published crystal structure of SLAC revealed that the two domain protein forms trimers in solution, where each trimer contains three trinuclear copper clusters (types 2 and 3) and three mononuclear copper centres (type-1) (Skálová *et al*., 2009). Site-directed mutagenesis was recently used to investigate the role of Tyr108 in the reduction of oxygen, and revealed the potential of this tyrosine residue to minimize the lifetime of reactive oxygen species by providing an additional source of reductant (Gupta *et al*., 2012). In the current study, 17 amino acids were individually mutated to Ala to identify the residues important for SLAC activity, as well as to confirm key residues involved in copper binding, and to predict those that could be targeted to alter the substrate specificity of this enzyme (Table S1, Fig. S5).

**Figure 3:**
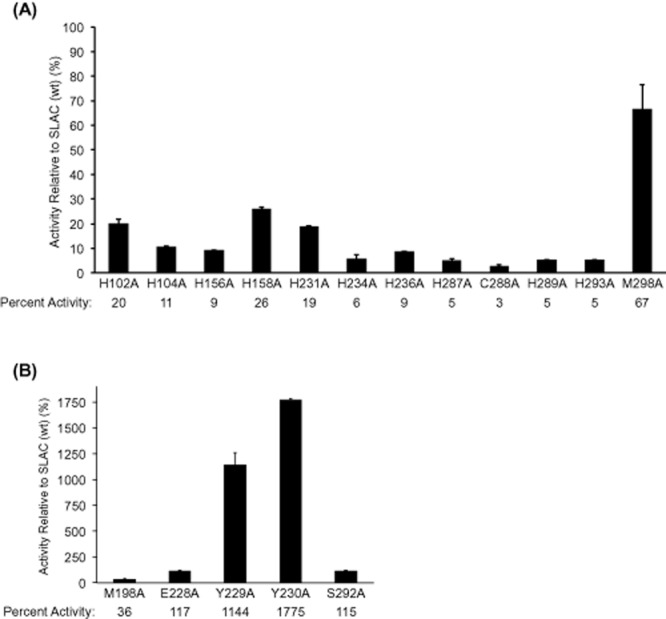
Percent activity of small laccase (SLAC) variants on ABTS relative to the wild-type enzyme.A. amino acid substitutions at positions that coordinate T1 and T2/T3 copper ions.B. amino acid substitutions at positions predicted to participate in substrate binding. The comparatively high activity of Y230A and Y229A mutants likely results from higher copper incorporation. Reactions (200 μl) contained 2 μg of enzyme prepared from standard cultivation conditions, 1 mM ABTS and 50 mM sodium acetate (pH 4.0). *n* = 3; errors indicate standard deviation.

Mutagenesis of SLAC confirmed an important role of the amino acid residues coordinating the mononuclear and trinuclear copper ions. Specifically, SLAC activity was dramatically reduced in mutant proteins containing 1 of 10 histidine substitutions or a cysteine substitution at positions predicted to coordinate the mononuclear (type-1) copper ion (H231A, C288A, H293A) and copper ions in the trinuclear (type 2 + type 3) cluster (H102A, H104A, H156A, H158A, H234A, H236A, H287A and H289A) (Fig. 3A). Substituting Met298 to alanine reduced SLAC activity by approximately 35%, which is consistent with role of this amino acid position in fine-tuning the redox potential of the type-1 copper ion (Durão *et al*., 2008b; Skálová *et al*., 2009). Notably, in a previous study of the corresponding Met502 residue in CotA, M502L and M502F substitutions reduced CotA turnover rates on ABTS to approximately 45% and 1% of wild-type levels, respectively, even though the redox potential of the mutant enzymes was higher than the wild-type (Durão *et al*., 2006). Similarly, the TvL laccase from *Trametes villosa* contains a Phe463 at the position typically occupied by the methionine that fine-tunes the redox potential of type 1 copper ions in other copper oxidases (Xu *et al*., 1997). When mutating Phe463 to methionine, the *k*_cat_ value of TvL with ABTS nearly doubles, despite the wild-type enzyme having higher redox potential (Xu *et al*., 1997). These studies indicate that future efforts to increase the redox potential of SLAC by mutating Met298 should consider detrimental effects such mutations could have on turnover rate.

Five of the 17 mutated proteins that retained or increased SLAC activity on 1 mM ABTS (pH 4) were predicted to form part of the shallow trimeric substrate-binding site at the surface of the enzyme (M198, Y229, Y230, E228 and S292) (Fig. 3B) (Skálová *et al*., 2009). In particular, Y229 and Y230 occupy most of the substrate-binding surface, and Y229A and Y230A substitutions increased SLAC activity on ABTS by more than 10 times compared with the wild-type enzyme prepared using standard growth conditions.

The copper content of Y230A was measured to determine whether the comparatively high activity of this variant was more likely the result of altered substrate binding or higher copper content compared with the wild-type enzyme. Indeed, the copper content in the Y230A mutant expressed under standard conditions was 2.3 moles of Cu^2+^ per mole of enzyme, 4.5 times higher than the wild-type enzyme expressed under aerobic conditions and twice that of wild-type SLAC produced under microaerobic conditions. Despite having higher copper content than wild-type SLAC produced using microaerobic conditions, the activity of Y230A with 1 mM ABTS was 1.2 μm min^−1^ mg^−1^, which is comparable to the activity of wild-type SLAC produced under microaerobic conditions. These results suggest that the Y230A substitution likely reduces SLAC activity on ABTS, but that increased copper binding can compensate for the loss in activity. In this case, further substitution of Y230 might identify residues that promote the cooperative loading of copper ions and production of fully activated forms of the enzyme.

While microaerobic cultivations conditions did not increase the copper content of the Y230A mutant, the molar ratio of copper to protein in S292A and E228A increased from 0.5 and 0.4 in standard preparations, to 1.5 and 0.9 in microaerobic preparations. In these cases, the increase in copper content also led to higher enzyme activities, where the activity of S292A on 1 mM ABTS at pH 4.0 increased more than 15 times (from 38 ± 0.2 nmol min^−1^ mg^−1^ to 670 ± 6 nmol min^−1^ mg^−1^), and the corresponding activity of E228A increased more than 4 times (from 34 ± 1 nmol min^−1^ mg^−1^ to 150 ± 6 nmol min^−1^ mg^−1^).

Similar patterns of copper incorporation between wild-type SLAC and the S292A mutant provoked an interest to further evaluate the substrate selectivity of S292A using compounds shown in Figure 1. Moreover, the similar increase in copper content of S292A and wild-type SLAC prepared from microaerobic conditions allowed more direct assessment of the amino acid substitution on substrate selectivity rather than copper binding. As in reactions containing wild-type SLAC, Na_4_Fe(CN)_6_ followed by polyaromatic compounds were transformed to highest extents after 20 min in reactions containing S292A (Fig. 1). However, kinetic parameters of S292A differed from wild-type SLAC. Specifically, the catalytic efficiency of S292A was slightly decreased on ABTS and slightly increased on 2,6-DMP (Fig. 2). Further, while the catalytic efficiency of wild-type SLAC was similar on quercetin, morin and myricetin, kinetic parameters could not be determined for S292A on quercetin and myricetin since the apparent *K*_m_ of S292A on these substrates exceeded substrate solubility (Fig. S6). Taken together, these results suggest that S292 localizes near the *meta* position of bound substrates. In this case, substitution of S292 to smaller amino acids is likely to promote enzyme activity on compounds with bulky functional groups at the *ortho* position, while substitution to larger polar amino acids would increase SLAC activity on polyaromatic compounds with un-occupied *meta* positions.

In summary, the observed activity on resveratrol, quercetin, morin, kaempferol and myricetin suggests that SLAC could be applied in organic syntheses involving bioactive phytochemicals, whereby corresponding compounds could be coupled to oligomeric forms or even grafted to pulp surfaces through free radical coupling. Mutation studies confirmed the critical role of all histidine residues predicted to directly coordinate copper ions at the enzyme active site, and indicate that saturating mutagenesis of Ser292, as well as Tyr229, Tyr230 and Met298, is a feasible approach to extending the catalytic efficiency of SLAC on low-cost mediators and bioactive compounds.

## Experimental procedures

### Materials

ABTS, 2,6-DMP, *p*-coumaric acid, *o*-coumaric acid, *m*-coumaric acid, caffeic acid, ferulic acid, sodium ferrocyanide (Na_4_Fe(CN)_6_), acetovanillone, phenol, syringaldehyde, syringic acid (4-hydroxy-3,5-dimethoxybenzoic acid), gallic acid (3,4,5-trihydroxybenzoic acid), 4-hydroxybenzyl alcohol, 3-(3,4-dihydroxyphenyl)-L-alanine (L-DOPA), 2,3-dihydroxybenzoic acid (2,3-DHB), quercetin, morin and rutin were purchased from Sigma. Kaempferol and myricetin were purchased from Extrasynthése. Resveratrol and apigenin were purchased from AK Scientific. Stock solutions (20 mM or 200 mM) were prepared in milliQ water, 100% ethanol (for ferulic acid, acetovanillone, syringaldehyde, 4-hydroxybenzyl alcohol, 2,3-DHB) or 100% DMSO (for *p*-coumaric acid, *o*-coumaric acid, *m*-coumaric acid, caffeic acid, gallic acid, syringic acid, resveratrol, apigenin, quercetin, morin, kaempferol, myricetin and rutin) and stored at 4°C.

### Gene cloning and protein purification

SLAC (SCO6712) was amplified using the polymerase chain reaction (PCR) starting from *S. coelicolor* genomic DNA purchased from the American Type Culture Collection. The PCR reaction was performed using the Pfx DNA polymerase (Invitrogen) and the following PCR cycles: denaturation at 95°C for 15 s, annealing at 53°C for 30 s, and elongation at 68°C for 1 min. PCR products were flanked by *Bse*RI restriction sites to facilitate cloning into p15Tv-L (GenBank accession EF456736). Ligation products (p15TvL_SLAC) were transformed into *E. coli* BL21(DE3) Gold strain (Stratagene). *E. coli* transformants were cultured at 37°C in a Terrific Broth medium containing 100 μg ml^−1^ of ampicillin and 1 mM CuCl_2_ as previously described (Zhang *et al*., 2001). Cultures were grown under standard aerobic conditions with shaking (200 r.p.m.) at 37°C to OD_600_ = 0.8–1.0, and protein expression was induced with 0.5 mM IPTG. After induction, the cells were incubated overnight at 16°C with shaking (aerobic conditions) or without shaking (microaerobic conditions). Cells were harvested after overnight growth, suspended in binding buffer (300 mM NaCl, 50 mM HEPES (4-(2-hydroxyethyl)piperazine-1-ethanesulfonic acid), pH 7.5, 5% Glycerol, 5 mM imidazole), passed through one freeze-thaw cycle, and then lysed by sonication. Cell extracts were cleared by centrifugation and incubated with Ni-affinity resin (Qiagen) for 2 h. The resin was then washed with 200 ml of washing buffer (300 mM NaCl, 50 mM HEPES, pH 7.5, 5% Glycerol, 30 mM imidazole) and eluted with approximately 10 ml elution buffer (300 mM NaCl, 50 mM HEPES, pH 7.5, 5% Glycerol, 250 mM imidazole). Protein concentrations were measured using the Bradford assay, and their purities were evaluated by 12.5% sodium dodecyl sulfate polyacrylamide gel electrophoresis. Purified proteins were dialysed against 10 mM HEPES-K buffer (pH 7.5) containing 0.3 M NaCl, flash frozen in liquid nitrogen and then stored at −80°C.

### Site-directed mutagenesis

Site-directed mutagenesis was performed using p15TvL_SLAC and the QuikChange site-directed mutagenesis kit from Stratagene (Brown *et al*., 2008). The sequences of primers used are shown in Table S1. Mutant SLAC proteins were overexpressed in *E. coli* BL21(DE3) and purified as described above for the wild-type protein. Transformants were cultivated in the presence of 1 mM CuCl_2_.

### Enzyme screening

The oxidase activity of SLAC was measured using the following substrates (1 mM concentration): ABTS, 2,6-DMP, *p-*coumaric acid, *o*-coumaric acid, *m*-coumaric acid, caffeic acid, ferulic acid, sodium ferrocyanide (Na_4_Fe(CN)_6_), acetovanillone, phenol, syringaldehyde, syringic acid (4-hydroxy-3,5-dimethoxybenzoic acid), gallic acid (3,4,5-trihydroxybenzoic acid), 4-hydroxybenzyl alcohol, L-DOPA, 2,3-DHB, resveratrol, apigenin, quercetin, morin, kaempferol, myricetin and rutin. The reactions (200 μl total volume) were performed at 60°C (20 min) in 0.2 ml strip-tubes with 50 mM universal buffer (pH 2 to 10; 50 mM acetic acid, 50 mM phosphoric acid, 50 mM boric acid) and 18.5 μg ml^−1^ enzyme. Absorbance readings were taken in a polypropylene 96-well plate (Greiner Bio-One). Reactions were performed in triplicate and were initiated by adding 180 μl substrate solutions to 20 μl enzyme; controls without enzyme were also included. Absorbance scans from 250 to 790 nm were performed for each reaction. Wavelengths and molar absorption coefficients used to measure oxidative activity were: ABTS, ε_420_ = 36 mM^−1^ cm^−1^ (Johannes and Majcherczyk, 2000); 2,6-DMP, ε_468_ = 14.8 mM^−1^ cm^−1^ (Machczynski *et al*., 2004); sodium ferrocyanide, ε_405_ = 0.9 mM^−1^ cm^−1^ (Machczynski *et al*., 2004); L-DOPA, ε_475_ = 3.6 mM^−1^ cm^−1^ (Laufer *et al*., 2006); syringic acid, 270 nm; caffeic acid, 330 nm; ferulic acid, 287 nm; resveratrol, 308 nm; quercetin, 385 nm (ε_385_ = 17.2 mM^−1^ cm^−1^ calculated from standard curve); morin, 385 nm (ε_385_ = 18.4 mM^−1^ cm^−1^ calculated from standard curve); kaempferol, 385 nm; and myricetin, 385 nm (ε_385_ = 15.9 mM^−1^ cm^−1^ calculated from standard curve). Compounds without extinction coefficient were followed by measuring substrate loss using a standard curve generated at the chosen wavelength.

### Kinetic analysis

Kinetic parameters were obtained at 60°C using 10 substrate concentrations (0.1 mM to 10 mM for ABTS and 2,6-dimethoxy phenol; 0.1 mM to 3.5 mM for quercetin; and 0.1 mM to 4 mM for morin and myricetin) at the optimal pH for each substrate. Reactions were initiated by adding substrate solutions to SLAC (final 125 or 150 μl reaction volume with 6 μg ml^−1^ SLAC), and initial rates were obtained by measuring product formation at 0, 1, 3, 5, 10 min. Kinetic parameters were calculated using the Michaelis–Menten equation (GraphPad Prism5 software; GraphPad Software, San Diego, CA, USA).

### Temperature and pH stability studies

Residual SLAC activity was measured after incubation at 75°C, 77°C, 80°C, 84.5°C, 90°C, 95°C, 98°C and 100°C for up to 15 h at pH 4.0. After pre-incubation, enzymes were cooled on ice, and residual activity was measured by incubating the enzyme sample for 20 min at 60°C with ABTS.

The effect of pH on SLAC stability was determined by incubating SLAC for up to 5 h at 22°C and pH 4 to 10. Universal buffer (50 mM acetic acid, 50 mM boric acid, 50 mM phosphoric acid) was used in these experiments. After pre-incubation, pH was adjusted to pH 4 and residual activity was measured by incubating the enzyme sample for 20 min at 60°C with ABTS.

### Metal analysis

Wild-type SLAC and mutant enzymes (1–3 mg) were dialysed using Pall polysulfone Macrosep ultracentrifugation tubes (10 kDa cutoff) into 10 mM HEPES buffer (pH 7.5) containing 0.1 M NaCl. Dialysed samples were acid digested using the MULTIWAVE microwave sample preparation method, and the metal content was determined using by Inductively Coupled Plasma Atomic Emission Spectrometry (ICP AES; Perkin Elmer Model Optima 7300DV ICP AEOS; Perkin Elmer, Waltham, MA, USA) at the Department of Chemistry of the University of Toronto as previously described (Högbom *et al*., 2005).

## Supporting information

Fig S1 Absorption spectrum of purified small laccase (SLAC).

Fig S2 Effect of temperature on small laccase (SLAC) activity.

Fig S3 pH Stability of small laccase (SLAC).

Fig S4 Chemical structures of natural bioactive phenolic substrates.

Fig S5 Multiple sequence alignment of small laccase (SLAC) and other small multicopper oxidases.

Fig S6 Michaelis–Menten plots for small laccase (SLAC) mutant S292A on quercetin and myricetin.

Table S1 Construction of small laccase (SLAC) mutants.
