# Supplementary material for: Biochemical studies of the multicopper oxidase (small laccase) from Streptomyces coelicolor using bioactive phytochemicals and site-directed mutagenesis
Source: Microb Biotechnol. 2013 Jul 1;6(5):588–97. doi: 10.1111/1751-7915.12068 (PMC3918160; doi:10.1111/1751-7915.12068)
Supplement: Fig S1 — Absorption spectrum of purified small laccase (SLAC). [file mbt0006-0588-sd1.pdf]

SUPPLEMENTAL FIGURE LEGENDS

Supplemental Figure 1. Absorption spectrum of purified SLAC. (A) The absorption spectrum of purified and concentrated SLAC (30 mg mL^{-1}; in 50 mM HEPES-K, pH 7.5, 0.5 M NaCl, 0.25 M imidazole, 5% glycerol) was recorded on a Varian Cary 50 spectrophotometer at room temperature. The wavelengths for the major peak and two shoulders are shown on the graph. (B) Corresponding image of the purified and concentrated SLAC preparation.

Supplemental Figure 2. Effect of temperature on SLAC activity. Reactions (200 μL) contained 3.7 μg of SLAC, 1 mM ABTS, and 50 mM sodium acetate (pH 4.0). n = 3; errors indicate standard deviation.

Supplemental Figure 3. pH Stability of SLAC. SLAC was pre-incubated for up to 5 h at room temperature in 50 mM universal buffer solutions (50 mM acetic acid, 50 mM boric acid, 50 mM phosphoric acid) ranging from pH 4.0 to 10.0. Reaction mixtures contained 1.2 μg SLAC, 1 mM ABTS, and were performed at 60 °C for 20 min. n = 3; errors indicate standard deviation.

Supplemental Figure 4. Chemical structures of natural bioactive phenolic substrates. Structures are shown for p-coumaric acid, caffeic acid, ferulic acid, apigenin and kaempferol.

Supplemental Figure 5. Multiple sequence alignment of SLAC and other small multicopper oxidases. The secondary structure of SLAC was assigned by DSSP and is shown above the

alignment. Similar resides are shadowed, and identical residues are boxed. SLAC residues that were mutated to Ala are indicated by filled arrows and numbered above the alignment. The proteins and the accession numbers are SLAC *Streptomyces coelicolor* copper oxidase (Q9XAL8), *Streptomyces griseus* EpoA (Q93HV5), *Saccharomonospora viridis* multicopper oxidase (C7MW31), *Nitrosomonas europaea* multicopper oxidase (Q82UE7), and *Clostridium beijerinckii* multicopper oxidase (A6LXP0).

Supplemental Figure 6. Michaelis Menten plots for SLAC mutant S292A on quercetin and myricetin. Enzyme activity corresponding to initial reaction rates was calculated for (A) quercetin and (B) myricetin at substrate concentrations up to the limit of substrate solubility. Reactions contained 6 μg mL^{-1} SLAC mutant S292A. Michaelis-Menten curves were plotted using GraphPad Prism5 Software.

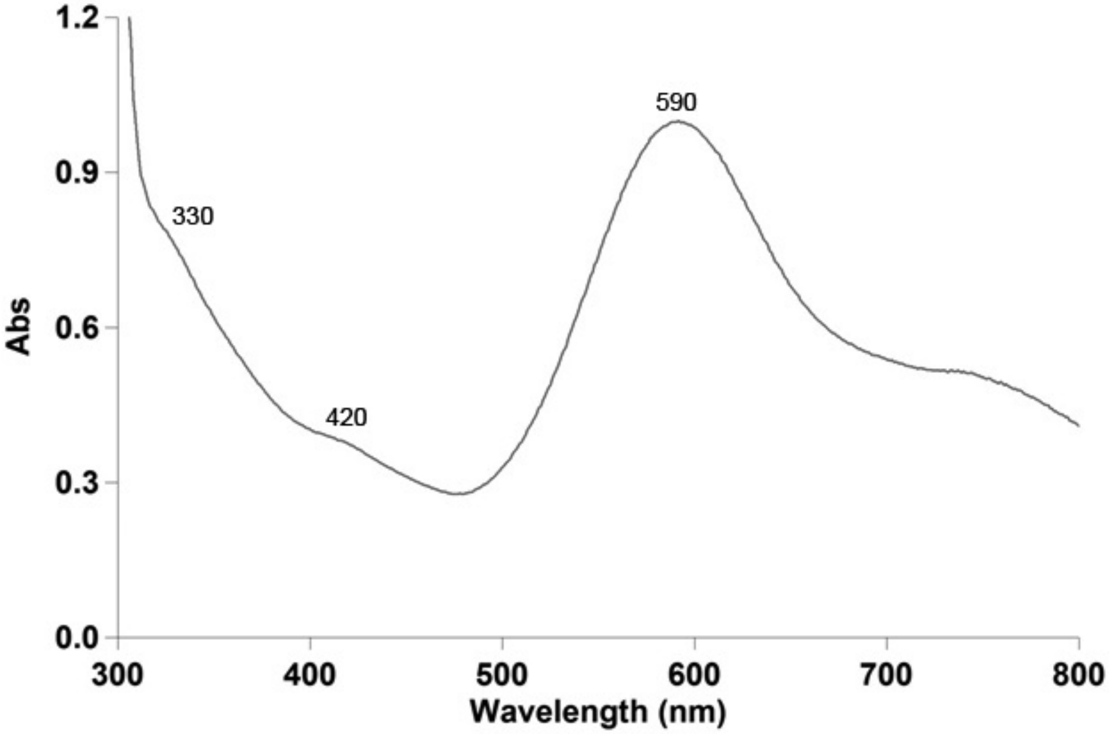

(A)

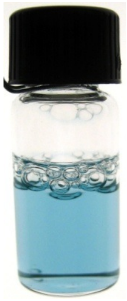

(B)

Supplemental Figure 1

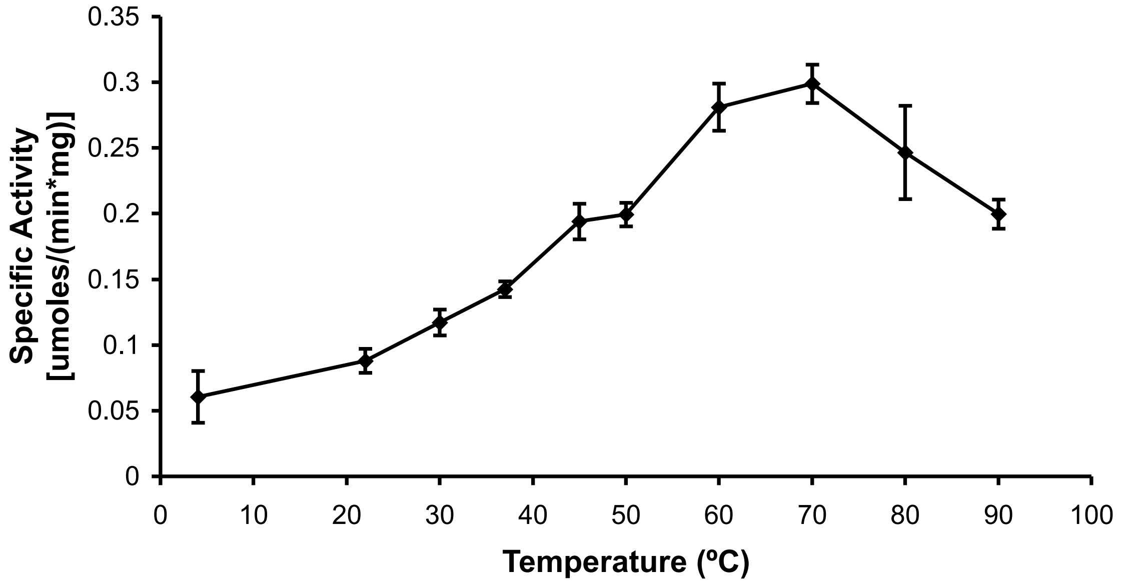

Supplemental Figure 2

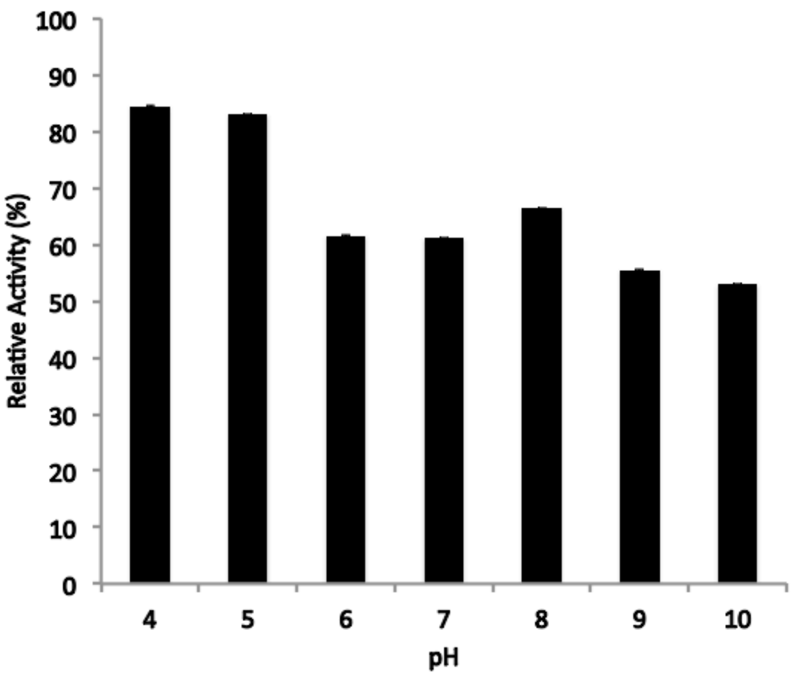

Supplemental Figure 3

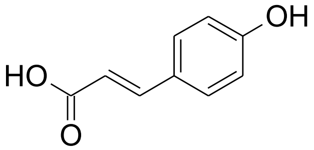

p-coumaric acid

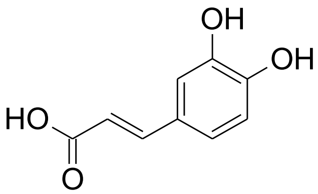

caffeic acid

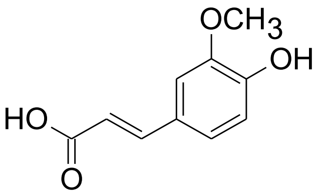

ferulic acid

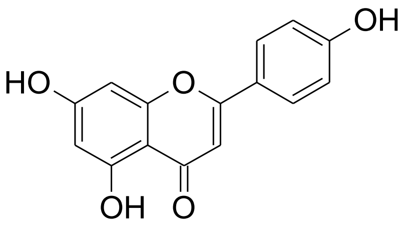

apigenin

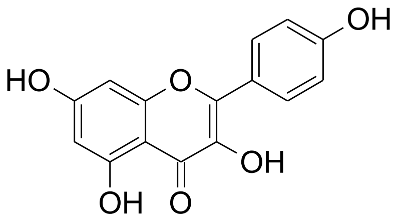

kaempferol

Supplemental Figure 4

```
SCO6712_laccase_Q9XAL8  -----------MDRRGFNRRVLLGGAAAATSLSIAPEVAGAAPAAKGITARTAPAGGEVR  49
EpoA_oxidase_Q93HV5     -----------MDRRTFSRRMLVGGAAAAATGVTSLSLGAVEASSAENPPRTAPAGGVVR  49
Svir_oxidase_C7MW31     -MIRKYARTGAFGKKVSRRSMLVGAAAGMAAPVVASTVGGTATAAPN--RAGDSQGEIR  57
NITEU_oxidase_Q82UE7    MHTRRNNNPFLYSCLTLFILIFLSLSSSLAQAGKVREYWIAAEKTDWNYAPTGANQIDLA  60
Cbei_oxidase_A6LXP0     -------------------MIITPDIQTLKFKMCKGVKYFELFAEQVEQEILPG-----  35

β1 α1 β2 β3 β4
SCO6712_laccase_Q9XAL8  -HLKMYAEKLADGQMGYG-FEKGKASVP----------GPLIEVNEGDTLHIEFTNTMDV  97
EpoA_oxidase_Q93HV5     -RLKMYAEKLPNGELGYG-FEKGKASIP----------GPLIELNEGDTVHIEFKNLTDV  97
Svir_oxidase_C7MW31     -RITMYAEELPGGMIGYG-LEPGKATVP----------GPVLEMWEGDTLEIELVNNTDH  105
NITEU_oxidase_Q82UE7    SDLGVWGKTLTYTKYRYIGYTDGSYSHPLPQPEWMGILGPTIRAAVGDTIKIHFLNKTDM  120
Cbei_oxidase_A6LXP0     ---------VFIKGWGYN------GSIP----------GPTIQVYPGDFVNIRVFNKLPE  70

β5 α2 β6 β7 β8   (102, 104)
SCO6712_laccase_Q9XAL8  RASLHVHGLDYEISSDGTAMNKS--DVEPGGTRTYTWRTHKPGRRDDGTWRP--------  147
EpoA_oxidase_Q93HV5     DASLHVHGVDYDIANDGTRMNKS--HVEPGGTRTYTWRTHAPGRRKDGTYEP--------  147
Svir_oxidase_C7MW31     RLSIHPHGVEYDTESDGSPLNDS--FNNPGERRTYIWRSRAPYQAKDGTWRP--------  155
NITEU_oxidase_Q82UE7    PLSMHPHGVMYDKDNEGADGGKGG-SIPPGERYTYTWIVDQDAGPGPGDP----------  169
Cbei_oxidase_A6LXP0     STSVHWHGLNVPNDMDGVPAVEPSPEISPGDYFDYRFKITNPPGTHMYHAHVNVVKQDMM  130

β9 α3 β10 β11   (155, 156, 158)
SCO6712_laccase_Q9XAL8  GSAGYWHYHDHVVGTEHGTGGIRNGLYGPVIVRRKGDVLPDAT------------HTIVF  195
EpoA_oxidase_Q93HV5     GSAGYWHYHDHVVGTDHGTGGIRKGLYGPVVVRRKGDILPDQT------------CTVVF  195
Svir_oxidase_C7MW31     GSAGYWHYHDHAMGTDHGTGGVLRGLYGALVVRRPGDILPDRQ------------YTIVF  203
NITEU_oxidase_Q82UE7    -SSIVWLYHSHVMAEEEVN----LGLIGTLIITAAGKAYSDKNPAPRDVDQEFIALYMIF  224
Cbei_oxidase_A6LXP0     GLLGGFVILNPNKTKVNKDYLLLMQEWHLVGLKKGEEVRPDIYKLDP--------FTNDF  182

β12 β13 β14 β15 β16   (198, 228, 229, 230, 231, 234, 236, 238)
SCO6712_laccase_Q9XAL8  N---------DMTINNRKPHTGPDFEATVGDRVEIVMITHGEYYHTFHMHGHRWADNRTG  246
EpoA_oxidase_Q93HV5     N---------DMMINNKTAHNSVNFEATVGDRLEFVMITHGEFYHTFHIHGHRWADNRTG  246
Svir_oxidase_C7MW31     N---------GMTINNRVAPDAPIFEARLGERVEFIAIGHGDHFHTFHLHAHRWADNRTG  254
NITEU_oxidase_Q82UE7    NEENDEESGLKHAINGRIFGNLSGFETRRGQRVRWHLVALGNEVDNHTVHWH----GQTV  280
Cbei_oxidase_A6LXP0     N---------MFTINGKSFPATTPMPIEYGDTVRLRLGAIQINHHPMHIHGHQFKVENAD  233

β17 β18 β19 β20 α4 β21   (284, 287, 288, 289, 292, 293, 298)
SCO6712_laccase_Q9XAL8  ILTGPDDPSRVIDNKITGPADSFGFQIIAGEGVGAGAWMYHCHVQSHSDM----GMVGLF  302
EpoA_oxidase_Q93HV5     ILTGPDDPSRVIGQQDLRPRDSYGLQIIAGERVGAGAWMYHCHVQSHSDM----GMAGLL  302
Svir_oxidase_C7MW31     LLSGPDDPTPIIDNKDLNPGSSFGFQVIAGEGVGPGAWMYHCHVQFHSDD----GMAGIF  310
NITEU_oxidase_Q82UE7    LDHG-----RRTDVVEIMPASMTSVDMVPRS---PGNWLFHCHVNDHMIA----GMATRW  328
Cbei_oxidase_A6LXP0     GNPLDKDNMILKNTILVASGETWDVIFKANN---PGVWPFHCHISHHMSNNLTKGTGGMF  290

SCO6712_laccase_Q9XAL8  LVKKPDGTIPGYEPHEHGG----ATAKSGESGEPTGGAAAHEHEH  348
EpoA_oxidase_Q93HV5     LIKKADGTIPGYEPHHAAGGTEKKAGAKGAGANADKAAKGAAEHQH  348
Svir_oxidase_C7MW31     LVRNEDGSLP-------------------PGAEEALERYRNHG-  334
NITEU_oxidase_Q82UE7    LVK------------------------------------------  331
Cbei_oxidase_A6LXP0     TTLVYTSNKSS-----------------------------------  301
```

Supplemental Figure 5

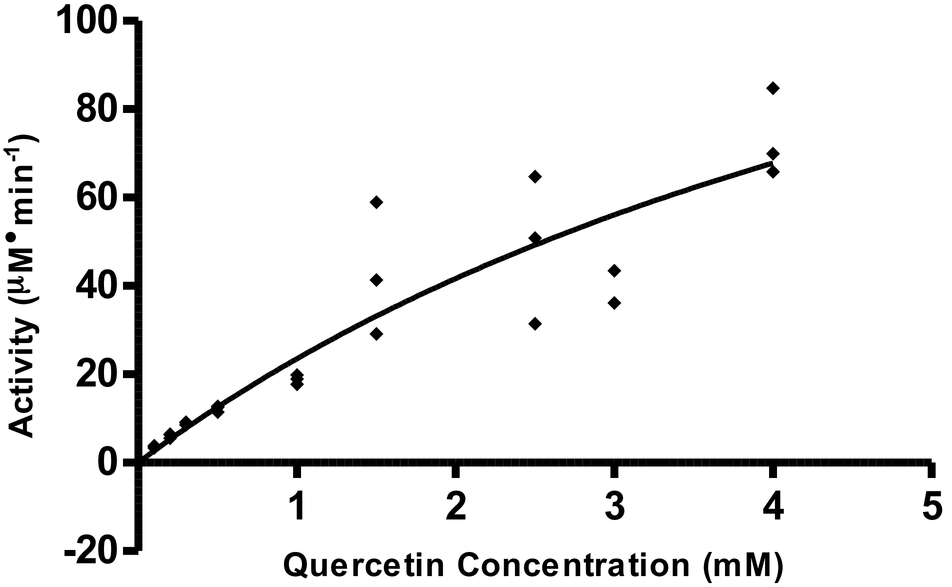

(A)

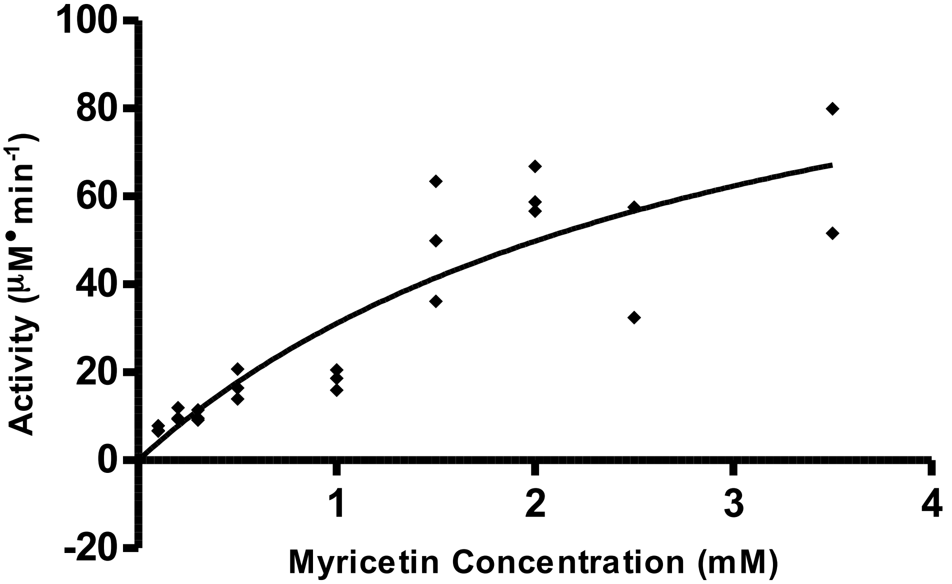

(B)

Supplemental Figure 6
