# Supplementary material for: Biochemical studies of the multicopper oxidase (small laccase) from Streptomyces coelicolor using bioactive phytochemicals and site-directed mutagenesis
Source: Microb Biotechnol. 2013 Jul 1;6(5):588–97. doi: 10.1111/1751-7915.12068 (PMC3918160; doi:10.1111/1751-7915.12068)
Supplement: Table S1 — Construction of small laccase (SLAC) mutants. [file mbt0006-0588-sd7.pdf]

Supplemental Table 1. Construction of SLAC mutants

Residue & mutation	Predicted function of mutated residues	Primers
H231A	type 1 Cu binding	CACGGGGAGTACTACGCCACCTTCCACATGCAC, GTGCATGTGGAAGGTGGCGTAGTACTCCCCGTG
C288A	type 1 Cu binding	GCGTGGATGTACCACGCCCACGTCCAGAGCCAC, GTGGCTCTGGACGTGGGCGTGGTACATCCACGC
H293A	type 1 Cu binding	CTGCCACGTCCAGAGCGCCTCCGACATGGGCATG, CATGCCCATGTCGGAGGCGCTCTGGACGTGGCAG
M298A	fine-tuning type 1 Cu redox potential	CACTCCGACATGGGCGCGGTGGGGCTGTTCCTG, CAGGAACAGCCCCACCGCGCCCATGTCGGAGTG
H104A	type 3 Cu binding	GCCAGCCTGCACGTGGCCGGCCTGGACTACGAG, CTCGTAGTCCAGGCCGGCCACGTGCAGGCTGGC
H156A	type 3 Cu binding	GGCTACTGGCACTACGCCGACCACGTCGTCGCC, GGCGACGACGTGGTCGGCGTAGTGCCAGTAGCC
H289A	type 3 Cu binding	GTGGATGTACCACTGCGCCGTCCAGAGCCACTCC, GGAGTGGCTCTGGACGGCGCAGTGGTACATCCAC
H158A	type 3 Cu binding	CTGGCACTACCACGACGCCGTCGTCGGCACCGAAC, GTTCGGTGCCGACGACGGCGTCGTGGTAGTGCCAG
H287A	type 3 Cu binding	GGGGCGTGGATGTACGCCTGCCACGTCCAGAGC, GCTCTGGACGTGGCAGGCGTACATCCACGCCCC
H236A	type 3 Cu binding	CACACCTTCCACATGGCCGGTCACCGCTGGGCG, CGCCCAGCGGTGACCGGCCATGTGGAAGGTGTG
H102A	type 2 Cu binding	CGTGCGGGCCAGCCTGGCCGTGCACGGCCTGGAC, GTCCAGGCCGTGCACGGCCAGGCTGGCCCGCACG
H234A	type 2 Cu binding	GTACTACCACACCTTCGCCATGCACGGTCACCG, GCGGTGACCGTGCATGGCGAAGGTGTGGTAGTAC
M198A	Substrate binding	GATCGTCTTCAACGACGCGACCATCAACAACCG, GCGGTTGTTGATGGTCGCGTCGTTGAAGACGATC
E228A	Substrate binding	CATGATCACGCACGGGGCGTACTACCACACCTTC, GAAGGTGTGGTAGTACGCCCCGTGCGTGATCATG
Y229A	Substrate binding	GATCACGCACGGGGAGGCCTACCACACCTTCCAC, GTGGAAGGTGTGGTAGGCCTCCCCGTGCGTGATC
Y230A	Substrate binding	CACGCACGGGGAGTACGCCCACACCTTCCACATG, CATGTGGAAGGTGTGGGCGTACTCCCCGTGCGTG
S292A	Substrate binding	CACGTCCAGAGCCACGCCGACATGGGCATGGTG, CACCATGCCCATGTCGGCGTGGCTCTGGACGTG
